# Locally induced neuronal synchrony precisely propagates to specific cortical areas without rhythm distortion

**DOI:** 10.1038/s41598-018-26054-8

**Published:** 2018-05-16

**Authors:** Haruo Toda, Keisuke Kawasaki, Sho Sato, Masao Horie, Kiyoshi Nakahara, Asim K. Bepari, Hirohito Sawahata, Takafumi Suzuki, Haruo Okado, Hirohide Takebayashi, Isao Hasegawa

**Affiliations:** 1 Department of Physiology, Niigata University School of Medicine, Niigata, Japan; 2 Department of Neurobiology and Anatomy, Niigata University School of Medicine, Niigata, Japan; 3 Research Center for Brain Communication, Kochi University of Technology, Kochi, Japan; 4 Department of Electrical and Electronic Information Engineering, Toyohashi University of Technology, Aichi, Japan; 5 Center for Information and Neural Networks, National Institute of Information and Communications Technology, Osaka, Japan; 6 Neural Development Project, Tokyo Metropolitan Institute of Medical Science, Tokyo, Japan; 7 Center for Transdisciplinary Research, Niigata University, Niigata, Japan

## Abstract

Propagation of oscillatory spike firing activity at specific frequencies plays an important role in distributed cortical networks. However, there is limited evidence for how such frequency-specific signals are induced or how the signal spectra of the propagating signals are modulated during across-layer (radial) and inter-areal (tangential) neuronal interactions. To directly evaluate the direction specificity of spectral changes in a spiking cortical network, we selectively photostimulated infragranular excitatory neurons in the rat primary visual cortex (V1) at a supra-threshold level with various frequencies, and recorded local field potentials (LFPs) at the infragranular stimulation site, the cortical surface site immediately above the stimulation site in V1, and cortical surface sites outside V1. We found a significant reduction of LFP powers during radial propagation, especially at high-frequency stimulation conditions. Moreover, low-gamma-band dominant rhythms were transiently induced during radial propagation. Contrastingly, inter-areal LFP propagation, directed to specific cortical sites, accompanied no significant signal reduction nor gamma-band power induction. We propose an anisotropic mechanism for signal processing in the spiking cortical network, in which the neuronal rhythms are locally induced/modulated along the radial direction, and then propagate without distortion via intrinsic horizontal connections for spatiotemporally precise, inter-areal communication.

## Introduction

Propagation of oscillatory spike firing activity with specific frequencies plays important roles in large-scale cortical networks^1–5^. In visual information processing, feedforward and feedback signals are conducted by the rhythmic activity of different frequencies^6–8^. Therefore, how such frequency-specific signals are induced and how the signal spectra of the synchronous rhythm are modulated during signal propagation are keys to understanding the principles of signal processing in the brain.

The tangential, inter-areal global connections mainly consist of axons from the pyramidal cells, while the radial, across-layer local connections consist of radially projected axons and dendrites of many types of pyramidal and non-pyramidal neurons. However, the functional roles of these different types of anatomical circuits in modulating neuronal spiking rhythms are not clearly understood. According to previous studies, following subthreshold electric stimulation, tangential propagation did not alter the frequency spectra of the signal^9^, whereas radial propagation specifically attenuated high-frequency signals^10^. Nevertheless, whether direction- and frequency-specific signal changes occur in the cortical signal processing accompanied with oscillatory spiking activity remains unclear. Moreover, active changes of the neuronal spiking rhythm, such as induction of frequency-specific synchronization, cannot be tested under the subthreshold stimulation condition. To the best of our knowledge, there has been no study in which a cell population is excited in a rhythmic manner, and radial- and tangential spectral change (frequency-specific reduction or induction of the neuronal spike rhythm) are simultaneously evaluated within the same tissue. It should be noted here that the tangential signal propagation must be non-linear presumably due to anisotropy of anatomical connectivity, although physiological validation of this view has also been difficult due to the lack of appropriate methods to regularly sample oscillatory neural activity from a wide area of active cortical circuits. This is why many previous studies employed subthreshold stimulation and simply regarded the brain tissue as a volume conductor.

**Figure 1:**
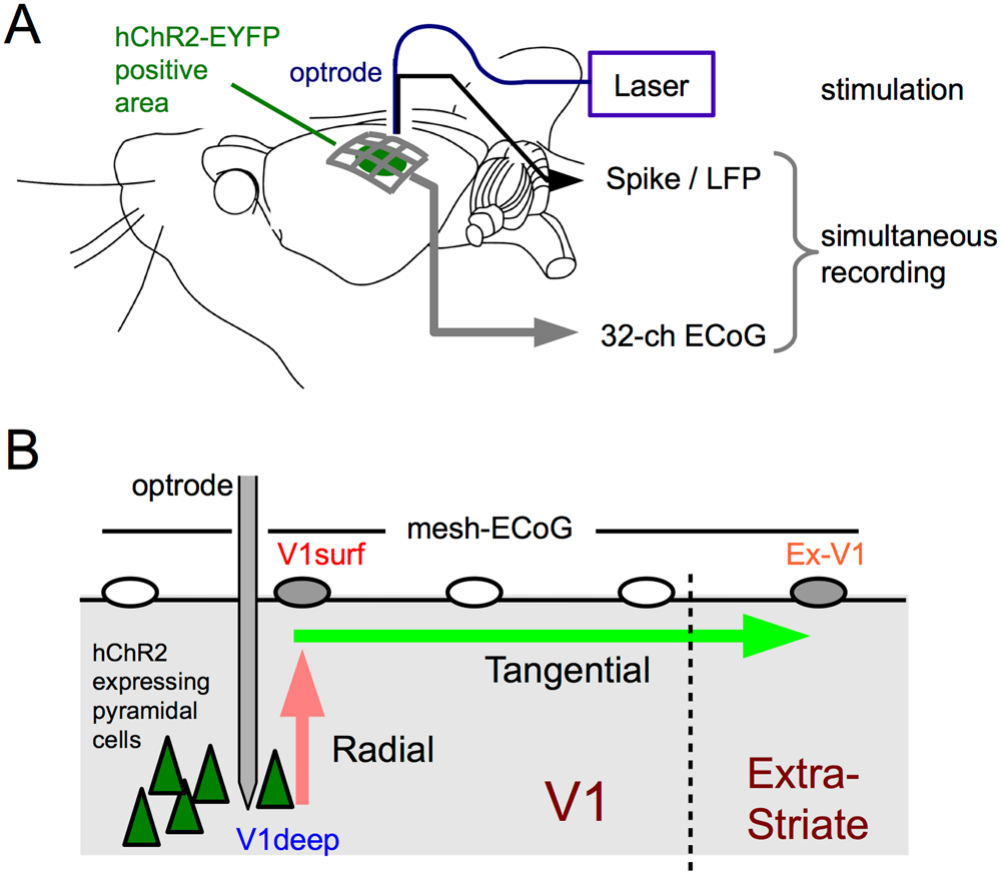
The experimental design. (**A**) The experimental setup. The AAV9 vector was injected at the infragranular layers of V1 and hChR2-EYEP was expressed under control of CaMKIIα promotor. Thus the transgene expression was mainly localized to deep-layer pyramidal cells. Photo-evoked neural activity was simultaneously recorded via a 32-ch mesh-electrocorticogram (mesh-ECoG) and an optrode (See Supplementary Table S1 for stimulation protocols). (**B**) A method for directly comparing spectral changes during radial- and tangential propagation. Spectral change during radial propagation was evaluated by subtracting the power spectrum of inctracortical LFP in V1 (V1deep) from that of ECoG recorded at the site nearest to the optrode (V1surf). Spectral change during tangential propagation was evaluated by subtracting the power spectrum of V1surf from that of ECoG recorded at the most responsive site distant by more than 2.2 mm from the optrode (Ex-V1). See Materials and Methods.

Here we hypothesize that radial- and tangential signal propagation are accompanied by different spectral changes even under the presence of spike activity. To test this hypothesis, we developed a new combination of optogenetics and micro-electrocorticogram (micro-ECoG)^11^ for simultaneous characterization of radial and tangential cortical propagation with oscillatory spike activity. We photostimulated^12–18^ infragranular excitatory neurons in the primary visual area (V1) of channelrhodopsin (ChR2)-transfected rats at a supra-threshold level (Fig. 1). Using both a depth electrode and a mesh micro-ECoG array^19–21^ in combination, we recorded intracortical local field potentials (icLFPs) at the stimulation site in the infragranular layers of V1 (V1deep), ECoGs at the cortical surface of V1 immediately above the stimulation site (V1surf), and ECoGs at responsive cortical surface sites far from the stimulation site in adjacent extrastriate visual areas (Ex-V1; see below). By applying photostimulation at various frequencies, we directly compared spectral changes during radial- and tangential signal propagation. Spectral changes during radial LFP propagation can be estimated by comparing the signals at V1deep and V1surf, while those during tangential signal propagation can be estimated by comparing signals at Ex-V1 and V1surf. To exclude the possibility that the effects of radial propagation might be confounded by the effects of comparing signals from different recording modalities, we also conducted additional experiments which evaluated the radial propagation effects by comparing signals at V1deep and V1surf recorded with the same recording system (Supplementary Fig. S1). Our analyses particularly focused on two classes of frequency components: the responses at the stimulation frequencies, and non-stimulation components which was not included in the stimulation frequencies or their harmonics.

## Results

### Expression of hChR2-EYFP in infragranular excitatory neurons

**Figure 2:**
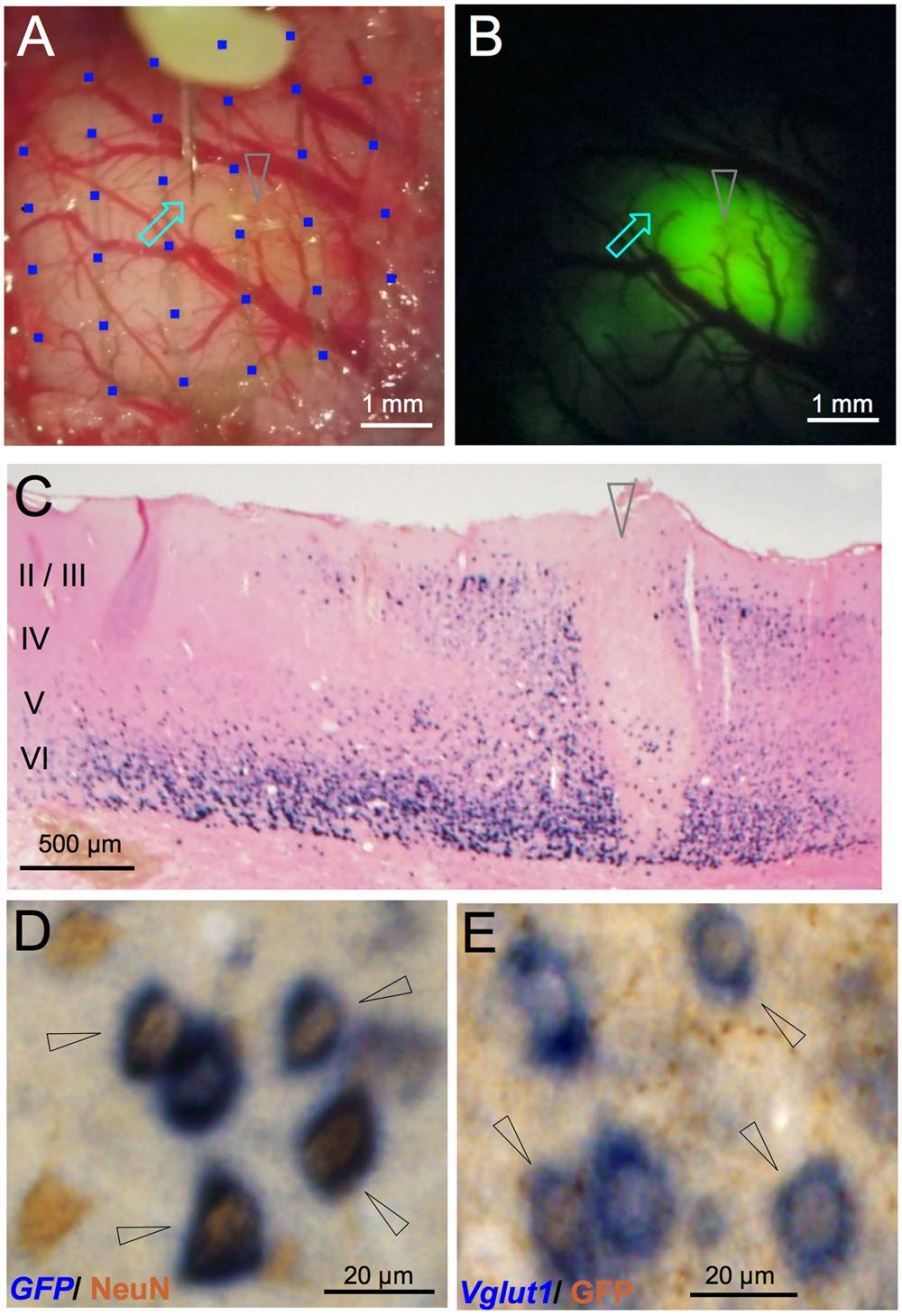
Microscopic and histological views of transgene expression. (**A**) A light field microscope view of V1. An optrode (arrow) inserted through a hole between the mesh-ECoG (blue squares) on V1. (**B**) A fluorescent microscope view of the same area as in A. EYFP fluorescence was observed around the injection site (arrowhead). The optrode insertion site (arrow) was within the fluorescence-positive area. (**C**) GFP immunostaining of a coronal section. The hChR2-EYFP fluorescence-positive cells were mainly observed in layers V and VI. Arrowhead indicates the injection site. Roman numbers indicate layers whose laminar borders were estimated from the result of *Vglut1 in situ* hybridization (ISH) of the adjacent section. (**D**) Double labeling for GFP and NeuN. Almost all ChR2-YFP expressing cells (detected by *GFP* probe, dark blue) were positive for NeuN, a mature neuron marker (detected by immunostaining, brown). (**E**) Double labeling for vGlut1 and GFP. Numerous ChR2-YFP-expressing cells (detected by GFP immunostaining, brown) were also positive for *Vglut1*, an excitatory neuron marker (detected by an ISH probe, dark blue) in the visual cortex. In D and E, double positive cells are indicated by arrowheads.

As shown in Fig. 1, a combination of micro-ECoG recording and optogenetics was introduced in this study. We used the CaMKIIα promoter to selectively express high-efficiency channelrhodopsin (hChR2)-EYFP in excitatory neurons^22^. We injected hChR2-EYFP expressing adeno-associated virus serotype 9 (AAV9) into the infragranular layers of V1 and observed EYFP fluorescence at the injected area (Fig. 2A,B) in all the hemispheres examined electrophysiologically (8/8). Numerous *EYFP-*positive cells were detected by *in situ* hybridization (ISH) mainly in the deep layers with weak spread to the other layers (Fig. 2C). The same layer specificity was reproduced in all the hemispheres examined by ISH. Almost all hChR2-EYFP-expressing cells showed immunoreactivity for NeuN, a mature neuronal marker (Fig. 2D). To examine whether hChR2-EYFP was selectively expressed in excitatory neurons, we next performed immunohistochemistry for GFP and ISH for *Vglut1*, a marker of excitatory glutamatergic neurons, on a single section. The GFP-immunoreactive neurons showed *Vglut*-positive ISH signals (Fig. 2E). These results show that hChR2-EYFP was induced mainly in infragranular excitatory neurons. We injected a small amount of the viral solution in additional experiments, in which the transgene-positive area was localized almost entirely within at the infragranular layers (Supplementary Fig. S1).

### Non-linear tangential distribution of photo-evoked ECoG signals

**Figure 3:**
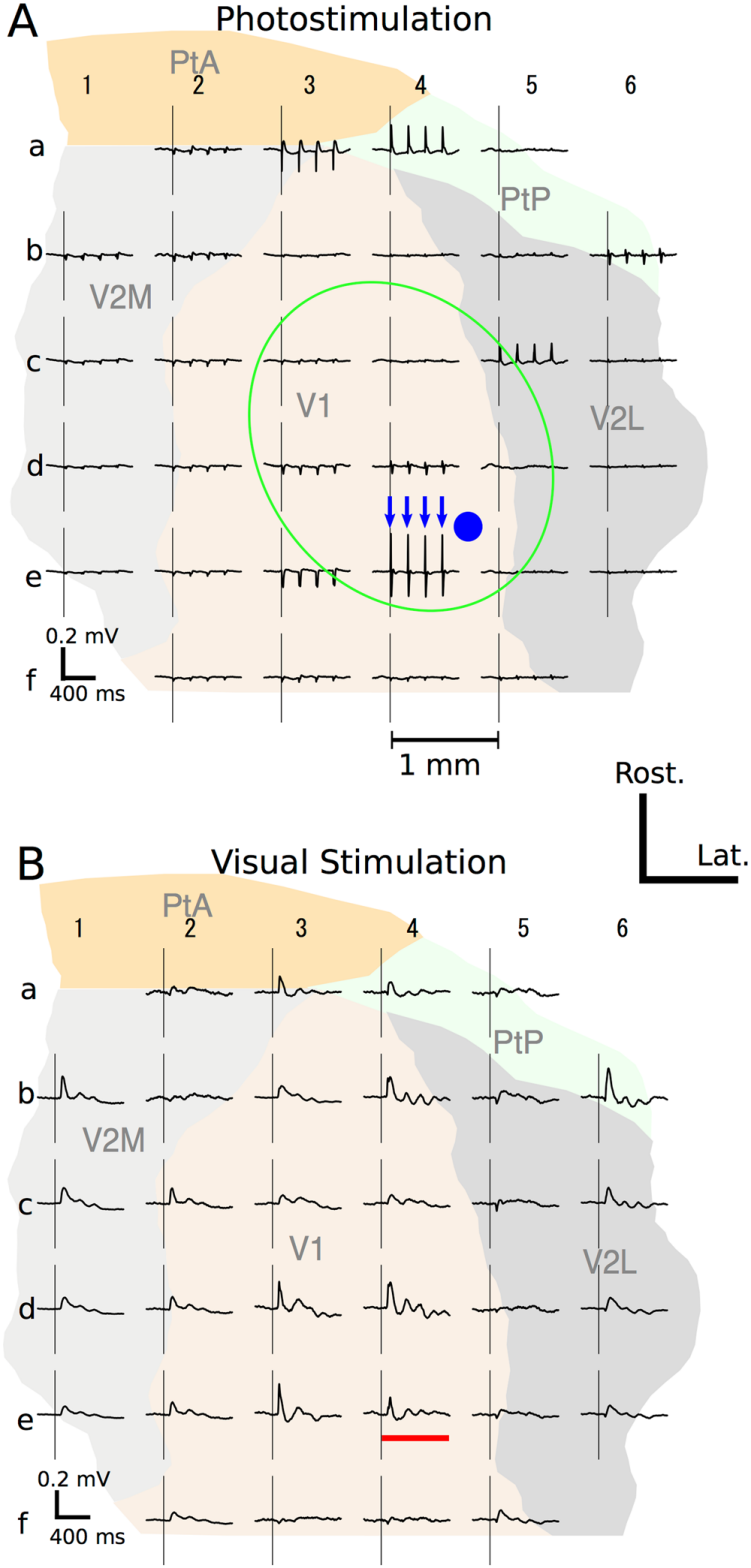
Cortical maps of photo-evoked (**A**) and visually-evoked (**B**) ECoGs. (**A**) Distribution of ECoG responses evoked by 5 Hz photostimulation recorded with the mesh-ECoG from the right hemisphere of a rat. Arrows indicate the timings of laser pulses. The gray ellipse indicates the approximate extent of the GFP florescence-positive area. The solid circle indicates the stimulation site. Pulse duration was 5 ms. The areal map on the background is adopted from the Paxinos and Watson’s Atlas^23^ and positioned according to the stereotaxic coordinates. V2M, secondary visual cortex, medial area; V2L, secondary visual cortex, lateral area; PtA, parietal association cortex; PtP, parietal cortex, posterior area. Each recording site on the mesh-ECoG is denoted by a combination of a number and a letter (*e.g*., 2d, 4a). (**B**) Visually evoked potentials (VEPs) recorded by the mesh-ECoG from the hemisphere as in A. Vertical lines indicate the onset of visual stimulation. Horizontal bar indicates the stimulus duration. In A and B, averaged data from 60 trials are shown.

Photostimulation via an optrode in the transgene-positive region elicited ECoG responses in V1 and surrounding cortical areas defined by Paxinos Atlas^23^ in all the hemispheres examined (8/8). As shown in Fig. 3A, every laser pulse reliably elicited event related potentials (ERPs) at multiple recording sites on the mesh-ECoG. The photo-evoked ERPs were largest at the recording site in V1 nearest to the optrode (in this example, column ‘4’ and row ‘e’, denoted as ‘site 4e’), but were also observed at extrastriate sites up to 3–4 mm from the optrode (*e.g*., sites 3a and 4a). The channel-wise response pattern of the photo-evoked ERPs was distinct from that of the visually evoked potentials (VEPs) obtained with the same recording settings (Fig. 3B). For example, the photo-evoked ERP at site 4e (Fig. 3A) was larger than the VEP (Fig. 3B), whereas the VEP was larger than the photo-evoked ERP at site 4d. The across-channel distribution of the photo-evoked responses (Fig. 3A) was more discontiguous or patchy compared to the VEPs (Fig. 3B). In some pairs of two adjacent sites (*e.g*., sites 3a-4a and sites 5c-6b, in Fig. 3A,B), the initial deflections of the photo-evoked ERPs were of opposite polarity to each other, while the initial deflections of the VEPs were basically positive in the entire recorded area. The polarity inversion and patchy activation of the photo-evoked ERP, observed in all the hemispheres examined, suggest involvements of local current sources in photo-activation of the visual cortices. In other words, photostimulation in the deep layers of V1 is not restricted locally but is affecting non-proximal surface sites in a manner that likely reflects intrinsic connectivity with circuit. From here on, we define the extrastriate site with the maximal VEP slope in each hemisphere as Ex-V1 (see Materials and Methods).

**Figure 4:**
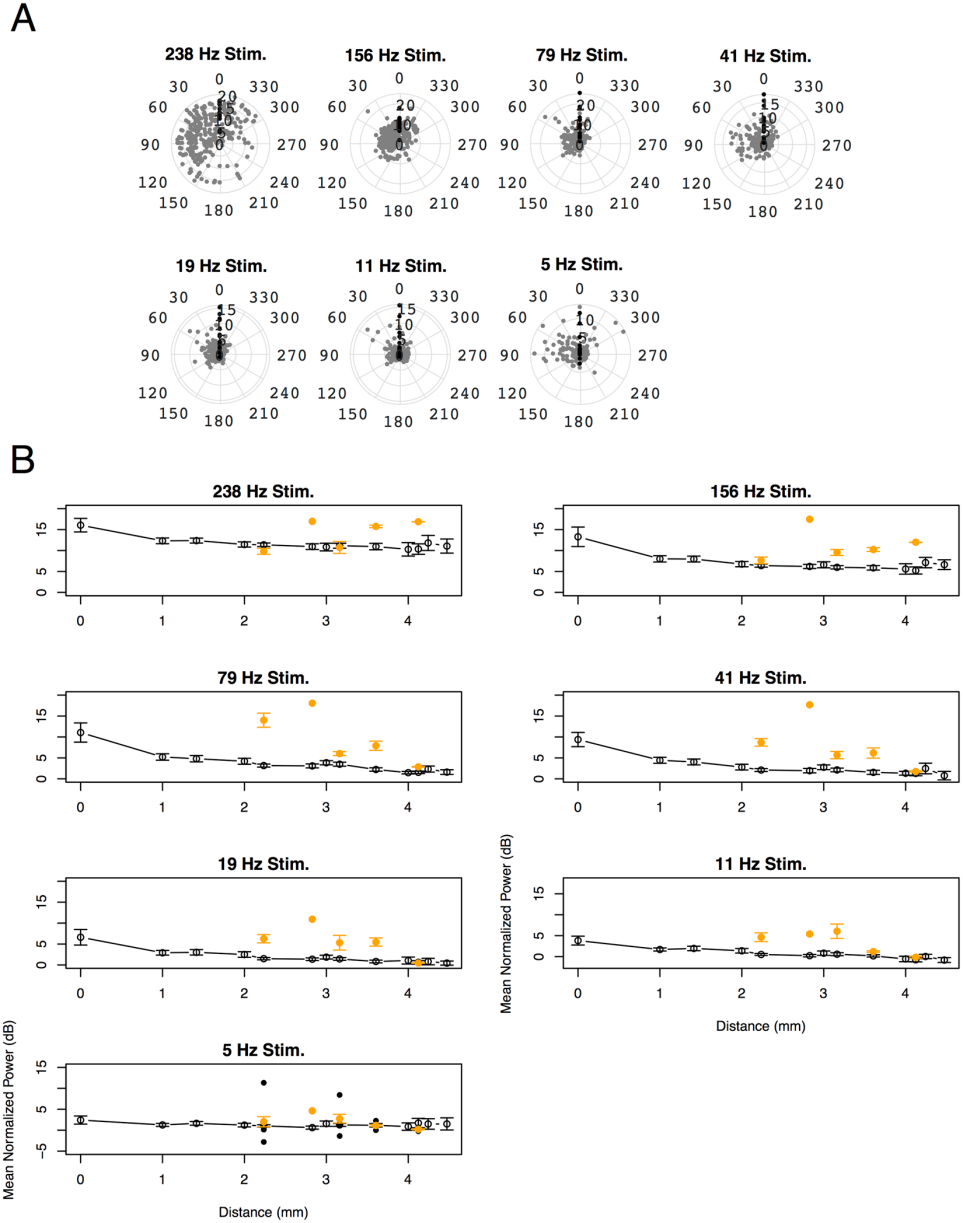
(**A**) A tangential anisotropy of cortical responses at 200 ms after the onset of photostimulation. Each dot represents an ECoG recording site (black: Ex-V1, gray: others). The angle indicates the deviation (deg) of the site from the V1surf−Ex-V1 line and the radius indicates mean normalized power at the site (dB). The deviation angle has a significant main effect in 156-, 79-, 41-, 19-, and 11 Hz stimulation (one-way ANOVA after sine transformation, *p* = 0.0007, 1.9 × 10^−10^, 2.0 × 10^−6^, 0.00011, and 0.0012, respectively), but not in 238- and 5 Hz stimulation (*p* = 0.10 and 0.48, respectively). (**B**) The normalized power recorded at each ECoG recording site is plotted against the distance from the corresponding V1surf site. The orange solid circles represent the data from Ex-V1 sites and the black open circles represent the data from the other ECoG sites. Error bars represent mean ± SEM. n = 16, 64, 64, 55, 101, 39, 32, 55, 41, 9, 18, 9, and 9 for the distance of 0, 1, 1.4, 2, 2.2, 2.8, 3, 3.2, 3.6, 4, 4.1, 4.2, and 4.5 mm, respectively.

These Ex-V1 sites located mainly in PtP and V2 (Supplementary Fig. S2). Tangential LFP propagation following 156-, 79-, 41-, 19-, and 11 Hz stimulation preferentially directed toward the Ex-V1 sites compared to other directions (Fig. 4A, one-way ANOVA after sine transformation, *p* = 0.0007, 1.9 × 10^−10^, 2.0 × 10^−6^, 0.00011, and 0.0012, respectively), though the direction specificity did not reach significance following 5- and 238-Hz stimulation (*p* = 0.48 and 0.10, respectively). As shown in Fig. 4B, the evoked power at most cortical surface decreased almost monotonically as the distance from the stimulation site increased, indicating non-linear, passive signal transmission is generally dominant in the tangential LFP propagation. By contrast, the response at many Ex-V1 sites distributed above this decay curve, exhibiting less signal reduction from V1surf. These results suggest that inter-areal functional connectivity is specifically strong between the V1 stimulation site and the Ex-V1 sites. In the following, we will focus on the differential power between Ex-V1 and V1surf for estimating the tangential spectral changes along intrinsic horizontal connections.

### Reliability of photo-evoked ECoG response

**Figure 5:**
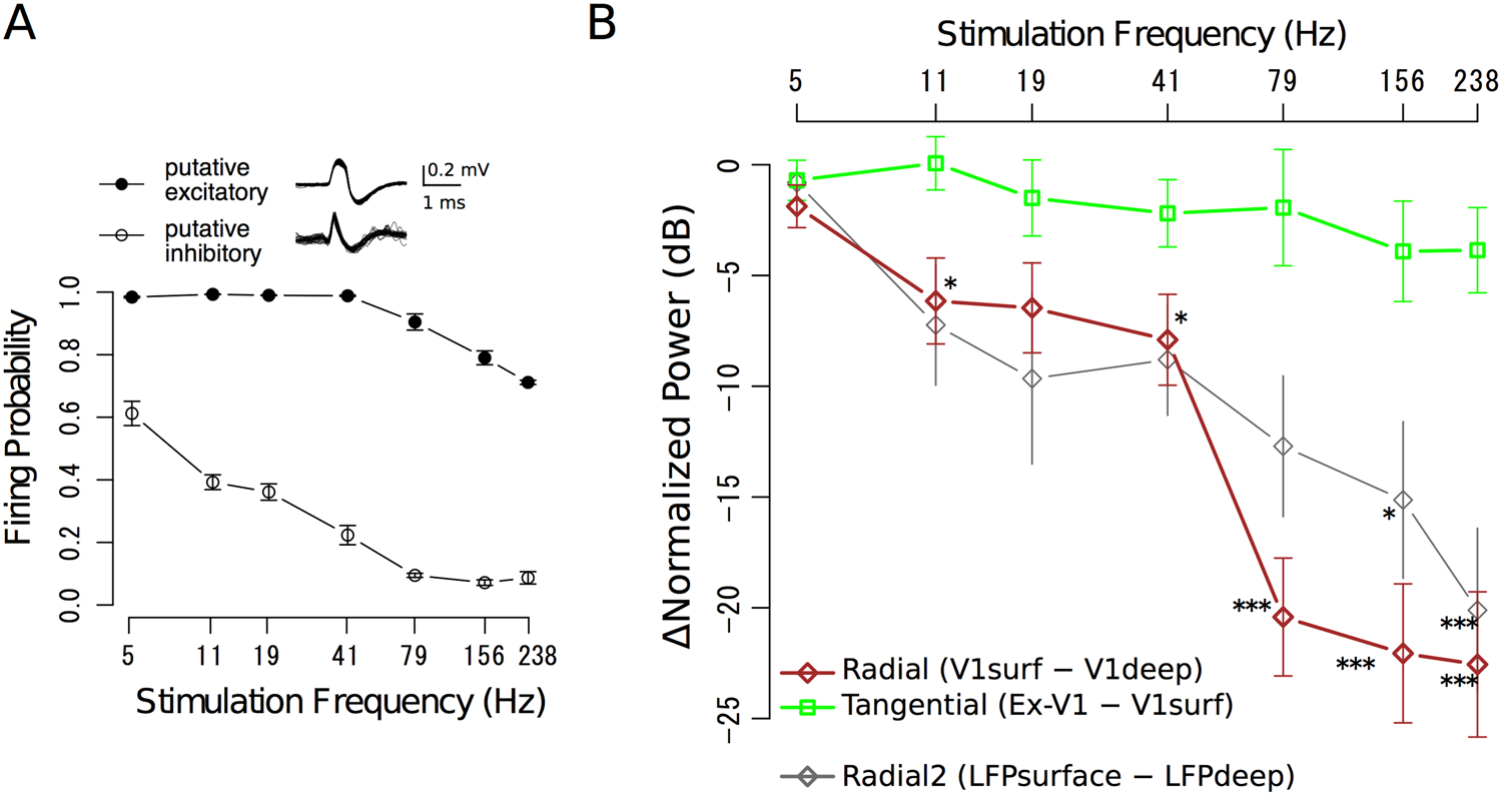
Frequency-response relationships at the stimulation frequencies. (**A**) Spike reliability of putative excitatory- (solid circle, n = 7) and putative inhibitory-neurons (open circle, n = 7) at various stimulation frequencies. Inset: superimposed traces of photo-evoked spike activity for 60 trials. (**B**) Reduction of fundamental LFP powers during radial (black diamond) and tangential (open square) propagation plotted against the stimulation frequency. Data were averaged over 16 stimulation sites. Gray diamonds (Radial2) represent the difference between the deep and surface probe tips of a multichannel optrode. *p < 0.05, **p < 0.001, ***p < 0.005, *post-hoc t-*test with Bonferroni correction after two-way ANOVA. See Supplementary Figure S6 for the normalized powers before subtraction. In A and B, error bars represent standard errors of mean (SEMs).

To evaluate the frequency properties of icLFP and ECoG signals, we photostimulated deep layer excitatory cells at various frequencies (Fig. 5). Photo-evoked responses of putative excitatory neurons^24–26^ as suggested by broad half-height spike width (0.33 ± 0.049 ms, mean ± standard deviation, Fig. 5A inset) were consistently observed after each laser pulse in response to all stimulation frequencies up to 41 Hz and with a probability of 0.7 even at 238 Hz stimulation (Fig. 5A, solid circle). These data indicate that hChR2-EYFP was introduced into deep-layer excitatory neurons of V1 as intended and that the spiking timing of these excitatory neurons was reliably controlled by the laser pulses. We also recorded from putative inhibitory neurons with narrower half-height spike width (0.11 ± 0.025 ms), but these neurons failed to respond to repeated laser pulses, particularly at higher stimulation frequencies (*e.g*., firing probability was around 0.1 when the stimulation frequency was 79 Hz or higher, open circle in Fig. 5A), suggesting that they were indirectly activated in our preparation.

As shown in Supplementary Figure S3, V1deep (blue trace) and V1surf (thick red trace, thin red traces display trial-wise results) responded to each laser pulse in a one-to-one ratio, even in experiments with “fast” frequency (*i.e*., higher than 100 Hz) stimulation. In the hemispheres lacking viral injection (Supplementary Fig. S4), the signal amplitude and power of V1deep during photostimulation were negligible compared to the transgene-positive hemispheres (amplitude: *p* = 3.9 × 10^−7^; power: *p* = 4.4 × 10^−5^, Student’s *t*-test). Likewise, photostimulation in the transgene-negative area of a virus-injected hemisphere evoked no photo-evoked neural responses (Supplementary Fig. S5). These results suggest that the one-to-one responses observed in this study were driven by photo-activation of hChR2-transfected neurons, not attributable to the contamination by optoelectric artifacts.

### Difference in signal attenuation between radial- and tangential LFP propagation

To evaluate changes in stimulus-driven LFP power during radial- and tangential propagation, we subtracted the fundamental components of LFPs at V1deep (blue line in Supplementary Fig. S6) from V1surf (red line), and V1surf from Ex-V1 (orange line, see Materials and Methods), respectively, at each stimulation frequency (Fig. 5B). The results show significant reduction of signal powers during radial propagation in all stimulation frequencies except 5 Hz, and the reduction was greater at the higher frequencies (brown diamond, *p* = 0.071, 0.0068, 0.0066, 0.0016, 1.4 × 10^−6^, 4.1 × 10^−6^, and 5.3 × 10^−6^ for 5, 11, 19, 41, 79, 156, and 238 Hz, respectively). By contrast, the attenuation of signal powers during tangential propagation from V1surf to Ex-V1 (green square) was much weaker and not significant even after high frequency stimulation (*p*-values were ranging from 0.96 for 11 Hz to 0.063 for 238 Hz). This dissociation is surprising because the inter-electrode distance between Ex-V1 and V1surf was at least 1.7 times larger than the distance between V1surf and V1deep. To rule out the possible effect of different recording systems for V1deep and ECoGs on the radial direction-specific high-frequency-dominant signal reduction, we also evaluated radial signal changes by comparing the LFPs recorded from the deep and surface probe tips of the same vertically aligned multichannel optrode (Supplementary Fig. S6, gray open squares and gray open circles for deep and surface, respectively). The results reproduced the significant high-frequency-dominant signal attenuation during the radial LFP propagation (LFP_deep_ *vs*. LFP_surface_: *p* = 0.032 and 0.0012 for 156- and 238-Hz stimulation, respectively, gray line in Fig. 5B). These results further confirm the radial direction-specific high-frequency-dominant attenuation (brown line in Fig. 5B).

It should be noted that, in the tangential plane, propagation of the photo-evoked powers was preferentially directed towards Ex-V1 when photo-stimulation was applied at 11–156 Hz (Fig. 4). Furthermore, as shown in Supplementary Fig. S6 (black open circles without lines), only weak responses were recorded even at the “on route” sites (sites between V1surf and Ex-V1). These findings suggest the non-linear nature of the tangential propagation, indicating that, unless boosted via the intrinsic anatomical connectivity, the tangential signal is simply propagating through the brain medium and undergoes passive low-cut filtering.

### Induction of transient low-gamma signals during radial LFP propagation

**Figure 6:**
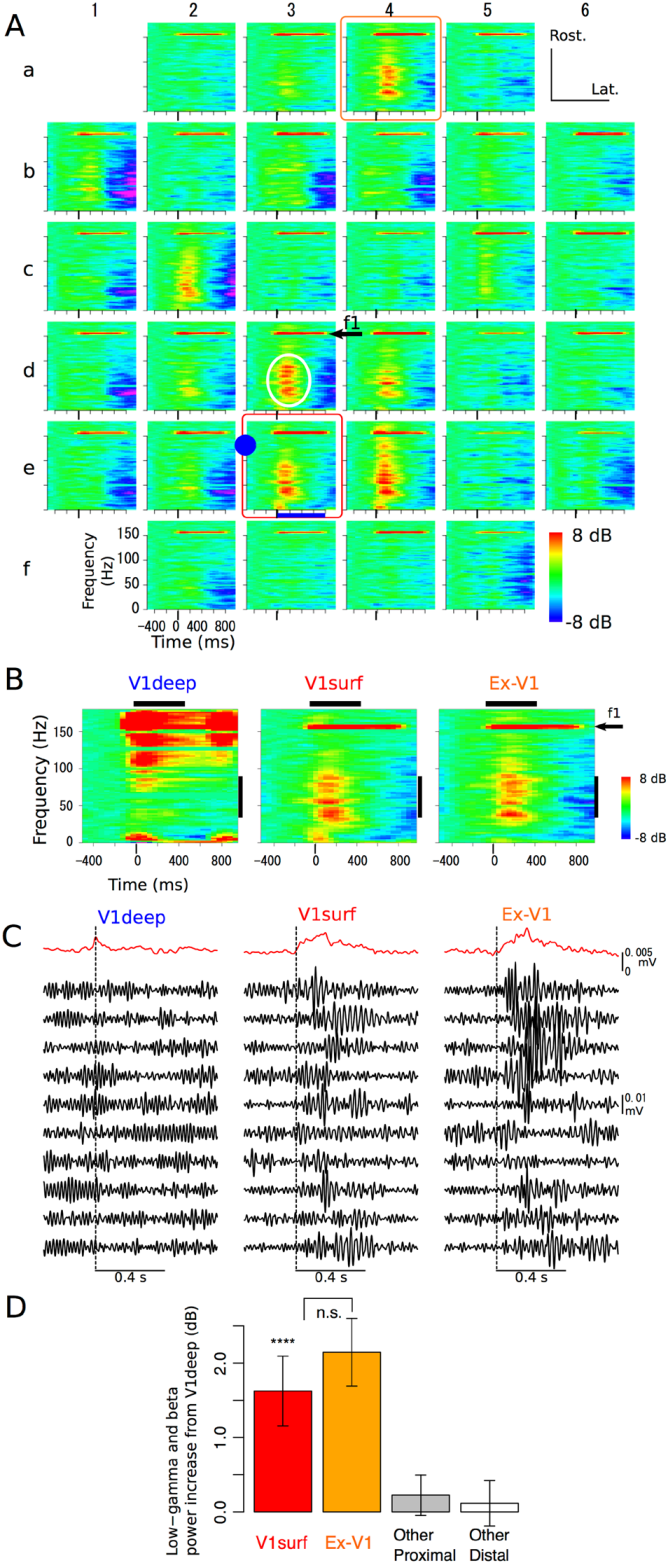
Low-gamma dominant rhythm induction during radial propagation. (**A**) Representative event-related spectral perturbations (ERSPs) of multichannel ECoG signals evoked by 156-Hz photostimulation via the optrode (solid circle). The responses at the stimulation (arrow, f1) and non-stmulation (white ellipse) frequencies were indicated at site 3d, for example. (**B**) ERSPs of simultaneously recorded at V1deep (left), V1surf (middle, indicated by red box in A), and Ex-V1 (right, indicated by orange box in A). Horizontal bars indicate the Hanning window for FFT used in power ratio analysis shown in D. Vertical bars indicate the frequency range of low-gamma band. (**C**) The low-gamma band signals simultaneously recorded at V1deep (left), V1surf (middle), and Ex-V1 (right). The black traces represent the trial-by-trial waveforms obtained from ten successive trials (the trials are same among V1deep, V1surf, and Ex-V1). The top red traces are the envelopes averaged over 60 trials. Vertical dashed lines indicate the onset of the stimulation period. The data are same as B. See Supplementary Figure S7 for the data with the other stimulation frequencies. (**D**) Low-gamma- and beta- band power induced at 200 ms after the stimulation onset. Difference in the total normalized power within beta or low-gamma bands between V1surf and V1deep (red), or Ex-V1 and V1deep (orange). Gray and white bars represent data from the other (*i.e*., not V1surf, nor Ex-V1) ECoG sites at distances of 1–2.2 mm and >2.2 mm, respectively. ****p < 0.005, paired-*t* tests.

Next we asked whether neuronal rhythms outside the frequency of the stimulation pulse train were induced during radial and/or tangential spike propagation. Following photostimulation at 156 Hz (Fig. 6A), non-stimulation LFP components (white ellipse at site 3d, for example) below the stimulation frequency (arrow) were observed at multiple ECoG channels. At V1deep (Fig. 6B, left), the non-stimulation components were mainly within the fast band (*i.e*., higher than 100 Hz). By contrast, the non-stimulation components of both V1surf (center) and Ex-V1 (right) were low-gamma- or beta-band dominant, suggesting radial-direction specific gamma-power induction. The trial-by-trial waveforms of the band-pass filtered signals clearly show low-gamma band oscillation induced by photo-stimulation in V1surf and Ex-V1 but not in V1deep with 41 Hz or higher frequencies (Fig. 6C and Supplementary Fig. S7). In pooled data (Fig. 6D), normalized power within beta- and low-gamma-band ranges at V1surf was significantly larger than that at V1deep (red bar, *p* = 0.0016, paired-*t*), whereas the difference between V1surf (red) and Ex-V1 (orange) was not significant (*p* = 0.27). These data suggested transient gamma-band rhythm induction during radial, but not tangential LFP propagation. Averaged beta- and low-gamma-powers were not significantly different from zero at proximal and distal surface sites other than Ex-V1, indicating that transiently induced beta- and low-gamma-powers are also non-linearly transmitted to specific cortical destinations along intrinsic connectivity.

For quantitative evaluation of beta- and low-gamma-rhythm induction relative to the evoked power at the stimulation frequency, we calculated the power ratios of the signals at V1deep, V1surf, and Ex-V1 200 ms after the stimulation onset (see Materials and Methods). In a representative experiment (Supplementary Fig. S8), the power ratios at V1surf (red) and Ex-V1 (orange) in gamma-band ranges were clearly higher than those of V1deep (blue). However, the differences between the power ratios of V1surf and Ex-V1 were not significant for all frequencies. The radial-direction-specific gamma-band rhythm induction was clearly observed in the pooled data (Supplementary Fig. S9). The low-gamma power ratio at V1surf (red) was significantly larger than at V1deep (blue) for all the stimulation conditions with 41 Hz or higher frequencies. The difference between the power ratios of V1surf and Ex-V1 (orange) was not significant for any combination of stimulation frequencies and response frequencies, suggesting lack of rhythm induction during tangential LFP propagation. The normalized low-gamma power induced by high-gamma- and fast-frequency stimulation was significantly larger (*p* = 0.0015, paired-*t*) at V1surf than at V1deep (Supplementary Fig. S10, Radial), while the difference between V1surf and Ex-V1 (Tangential) was not significant (*p* > 0.75). These data suggest that induction of non-stimulation components in low-gamma bands is associated with the radial-, rather than tangential-, LFP propagation.

## Discussion

### Radial/tangential anisotropy of cortical spike synchrony propagation

We developed a new combination of optogenetics and micro-ECoG recording for simultaneous characterization of radial- and tangential cortical propagation of LFP signals with oscillating spike activity, and showed direct evidence for non-linear neuronal rhythm propagation in the tangential plane as well as for radial-direction-specific spectral changes. In the analyses of the stimulating frequency components of the signals (Fig. 5), the radial propagation exhibited substantial signal reduction especially at high frequencies, which was not significant in tangential propagation to Ex-V1. Moreover, the analyses of non-simulation components (Fig. 6) further suggested that low-gamma dominant rhythms might be specifically induced during radial propagation. Such an anisotropy was observed even though the length of inter-areal tangential propagation from V1surf to Ex-V1 was longer than the length of intra-areal radial propagation from V1deep to V1surf in the present study.

**Figure 7:**
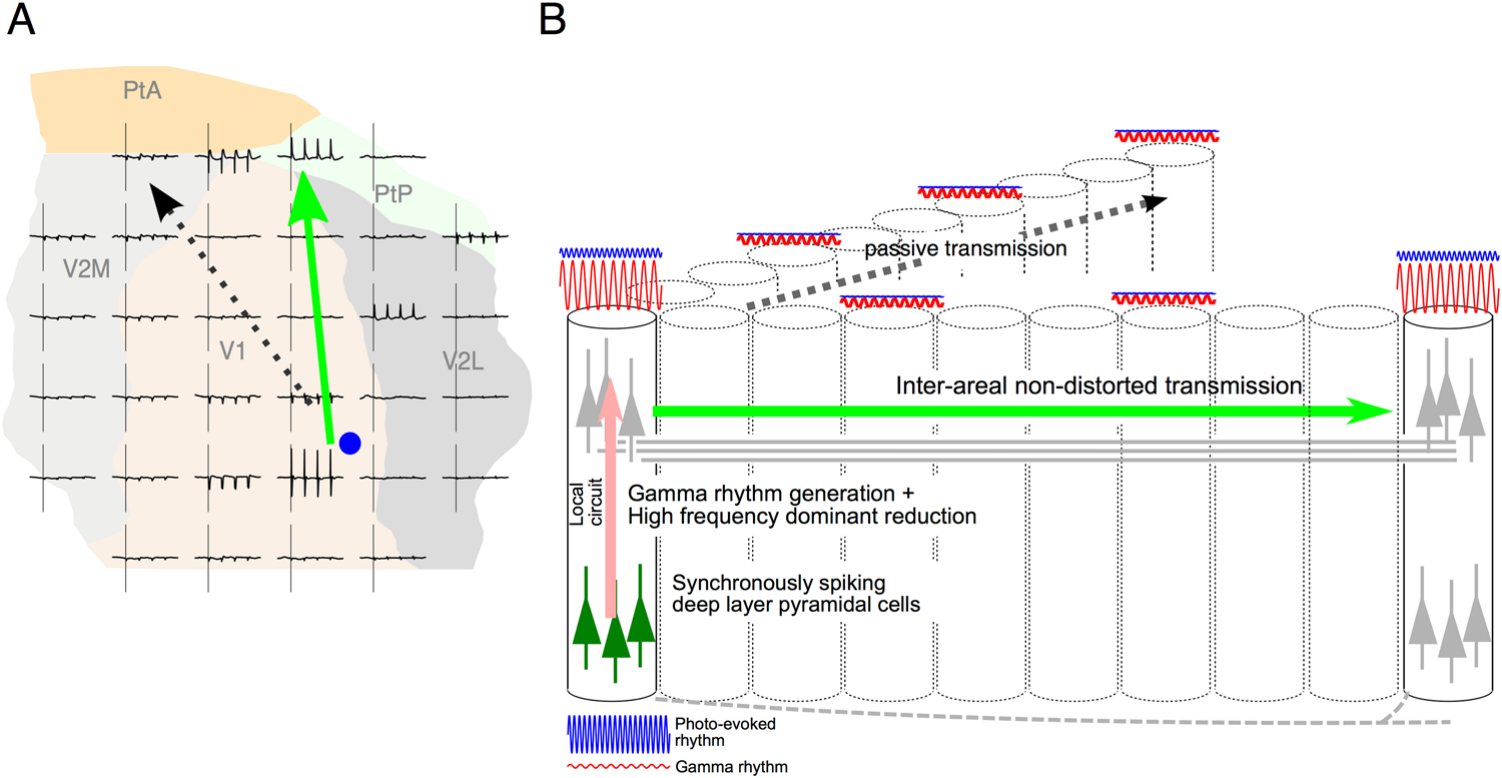
Schematic illustration of induction/transfer of oscillatory neuronal rhythms along the radial and tangential signal propagations from a planer (**A**) and three-dimensional (**B**) views. Synchronously photostimulated infragranular pyramidal neurons activate the local across-layer circuits where high-frequency neuronal rhythm is attenuated and the low-gamma dominant neural rhythms are induced. The locally modulated signals are conducted to other cortical areas via region-specific inter-areal tangential networks (green solid arrows in A and B), which have flat frequency properties and are accompanied by less power reduction. Without an intrinsic inter-areal connection, tangential LFP propagation is conducted passively (black dotted arrows).

In previous studies, membrane potential fluctuations of pyramidal cells, either spontaneous or evoked by subthreshold electric stimulation, were used as signal sources for evaluation of spectral changes^9,27^. By contrast, we stimulated deep layer pyramidal neurons of V1 by suprathreshold laser pulses. Therefore, radial-direction-specific signal attenuation and low-gamma power induction found in this study may be ascribed not only to the electro-tonic, passive properties such as the cable properties of apical dendrites of individual deep-layer pyramidal cells^27^, but also to the multi-synaptic local circuits which consist of photostimulated deep-layer pyramidal cells and indirectly driven interneurons and supragranular pyramidal cells (Fig. 7B, the leftmost column). On the other hand, preservation of neuronal spiking rhythms over 2.2 mm along the tangential direction to Ex-V1 without any significant high-frequency spectral reduction is presumably due to a population of cortico-cortically projecting axons firing in a synchronous manner (Fig. 7, horizontal green lines). Theoretically, flat frequency properties might also be explained by propagation via the cerebrospinal fluid. This possibility, however, is not likely in our preparation since current conduction via pure resisters cannot explain the patchy spatial pattern and phase-inversion of the photo-evoked ERPs (Fig. 3A). The patchy spatial response pattern of the photo-evoked ERP responses is incompatible with another theoretical possibility that the LFPs propagated regeneratively within infragranular or supragranular layers, though a tangential “leap” propagation of neuronal rhythms via the infragranular layers or white matter cannot be ruled out by the present study (Fig. 7, dashed line). Rather, site-specific “leap” inter-areal propagation in the tangential plane (Fig. 4) likely reflects projections from V1 to the retinotopically corresponding parts of extrastriate areas^28–30^. Weak responses in “on route” sites (sites between V1surf and Ex-V1) at stimulating frequency (black open circles without lines in Supplementary Fig. S6) and weak low-gamma- or beta-band power generation at non-Ex-V1 sites (gray and white bars in Fig. 6D) provide additional evidences for the spatial specificity of inter-areal LFP propagation. In 238-Hz stimulation, the anisotropy of LFP propagation in the tangential plane was less evident (Fig. 4A). It is possible that non-physiological circuit hyperactivity occurred in these cases due to excessively high stimulation frequency, and/or that the “on route” ECoG electrodes captured leak currents via the membrane capacitance of projecting axons.

### Possible roles of direction-specific LFP modulation in inter-areal cortical networks

In this study, transgene expression was mainly observed in excitatory neurons at the deep layers of V1 (Fig. 2C–E). The deep-layer pyramidal cells receive feedback signals from higher areas through the soma and apical dendrites in the layer I, and they send feedforward and feedback signals to other cortical areas^31–33^. Based on our findings, we propose an integrated model explaining the transfer and synthesis of oscillatory neuronal activity through tangential and radial cortical circuits (Fig. 7). Basically, cortico-cortical signal conduction via specific horizontal networks accompanies little spectral changes, which is relevant to undistorted signal transmission and precise inter-areal spike synchronization. Infragranular pyramidal neurons receive these cortico-cortical inputs, and then relay the activity to the local across-layer circuits, where neuronal rhythms of deep-layer origin are attenuated, especially in high-frequency bands, and the low-gamma dominant neural rhythms are induced. The locally modulated signals, in turn, are conducted to other cortical areas via specific inter-areal networks again. Even though rodent V1 is believed to be deficient in anatomical homologues of cortical columns, it is possible that the aforementioned principle might be remotely related to the principle of cerebral cortical information processing in mammalian species other than rodents where the functional columns play a key role as network hubs of inter-areal interactions.

### Cortical circuits underlying photo-induced gamma-power generation

Following suprathreshold stimulation of deep-layer excitatory cells, we observed induction of the non-stimulation LFP components during radial propagation that could not be solely explained by the stimulus pulse train itself (Fig. 6). This finding is consistent with a theoretical study suggesting that recurrent neuronal interaction within V1 together with feedback from the extrastriate cortex plays an important role in generating gamma-band signals in V1^34^. An important contribution of inhibitory local feedback signals from the parvalbumin (PV) neurons to the pyramidal neurons in gamma-band power generation has been proposed in previous studies. For example, Sohal *et al*. reported gamma-band power generation following a single-pulse photostimulation of the infragranular pyramidal cells, and found the generation of gamma-power was significantly reduced by photo-inhibition of the PV-positive cells^35^. Though we did not directly test involvement of PV neurons in gamma-rhythm induction, our data are consistent with this study assuming that photo-activated infragranular pyramidal cells drove inhibitory interneurons in our study. Moreover, involvement of the cortical inhibitory circuits might explain the late negativity of the ECoG responses after prolonged photostimulation in our study (Fig. 6A,B).

Spiky changes of the averaged ERP waveform are observed in response to individual photo-stimulation pulses (Supplementary Fig. S3). Thus, it may appear paradoxical that stimulus-evoked gamma oscillation cannot be visible as any change in the averaged waveform (*e.g*., site 4e in Fig. 3A) whereas event-related spectral perturbations (ERSPs) in many frequency bands outside of the stimulation frequency are observed in the time-frequency plot (Fig. 6A,B). For two reasons, however, we regard the lack of averaged ERP changes as evidence indicating that the recorded gamma rhythm was indirectly induced, rather than directly evoked by optogenetic stimulation. Firstly, gamma activity was more evident at V1surf and Ex-V1 than V1deep, the nearest recording site where the directly evoked neural responses should be maximal. Secondly, the enhancement of low-gamma powers is visible by eye in trial-by-trial traces of the filtered signals (Fig. 6C and Supplementary Fig. S7). Apparent dissociation of ERSP and the averaged ERP waveform was not unique to our study but repeatedly described in previous literature reporting that whereas early stimulus-evoked oscillation is recognized as a change in the averaged ERP waveform, indirectly induced oscillation is usually asynchronous to stimulus onset and thus does not cause waveform changes following trial average^36,37^. Therefore, even though the low-gamma and beta rhythm observed in the ERSP was 400–800 ms in duration, we speculate that the oscillation was indirectly induced, not directly evoked by photostimulation.

### The spread of the optogenetically activated area

As shown in Fig. 3, the VEPs of similar size were found in four neighbor V1 channels corresponding to the upper-nasal quadrant visual field in which visual stimulation was presented (Fig. 3B, sites 3d, 3e, 4d, and 4e). By contrast, optogenetically-evoked responses were not pronounced in the V1 channels nearest to the best-responding channel (Fig. 3A, site 4e), with the second best response observed at a remote Ex-V1 site (site 3a) away from the optrode. These findings suggest that the cortical spread of the direct optogenetic activation is as restricted as 1 millimeter or less. Han *et al*.^38^ previously reported that the radius of the photostimulated area was 1.2 mm when laser power was 80 mW/mm^2^ (2.4 mW at the fiber tip in their experimental setup). In our experiments, the spread of direct photo-excitation may be more restricted because we used a weaker laser power (0.7–2.2 mW). Appropriate stimulation power to drive sufficient but not excess number of neurons would likely play a key role both in destination-specific oscillatory signal propagation along the horizontal connection (Fig. 4) and transient gamma power induction within the local cortical circuit (Fig. 6C, Supplementary Figs S7 and S9). The high spatial specificity of channel-wise photo-evoked ERP patterns (Fig. 3A) also provides evidence showing that micro-ECoG can faithfully capture the profile of the underlying signal source at a high spatial resolution of 1 millimeter or less.

In the main experiments, some cells in the superficial layers were also transfected, likely due to back flow of the viral solution. However, we believe that this contamination does not weaken our conclusions for three reasons. Firstly, the supragranular layers might not receive laser power directly because the tip of the optical fiber was advanced to the infragranular layers and the light flux was emitted mainly downwards in our study. Secondly, even if some superficial cells were directly photo-activated, then the photo-evoked signals from these cells should lead to an underestimation, rather than overestimation, of the spectral changes between V1surf and V1deep. Thirdly, we performed additional experiments with much smaller injection volumes (200 nL, 1/5 of the main experiments), which reproduced the radial-direction spectral changes (Fig. 5B and Supplementary Fig. S6, gray lines) found in the main experiments.

### Advantages of combined micro-ECoG and optogenetics

Optogentics allows us cell-type-specific, pathway-specific, and frequency-specific stimulation of synchronous neuronal rhythms at a suprathreshold level without large electrical artifacts. These features are unachievable by conventional intracortical microstimulation. A number of studies have recorded the distribution of photo-evoked neural activity using metal microelectrode arrays (MEAs)^39^ or functional magnetic resonance imaging (fMRI)^40–43^, but the time resolution of fMRI and scalability of MEAs are limited. In this study, we observed a strictly time-locked response and intrinsically induced gamma rhythm at sites distant from the stimulation site by 2.2 mm or larger even at frequencies over 100 Hz.

It was traditionally believed that the electrode impedance and shape affect the sensitivity of single unit recording^44,45^. However, for LFP and EEG, effects of these parameters on recording quality remain unsettled because of larger signal source size associated with LFP and ECoG recordings^46,47^. Importantly, the radial-direction-specific spectral changes found between intracortical LFP (V1deep) and ECoG signals in our study were not solely ascribed to the impedance difference between the recording modalities, because the conclusion was further confirmed by subtracting deep and surface LFPs recorded via probe tips with the same impedance and shape (Fig. 5B and Supplementary Fig. S6). A preliminary experiment suggests that the frequency-specific attenuation in radial propagation occurred mainly within the infragranular layers (Supplementary Fig. S11).

The photostimulation efficiency of putative excitatory cells (almost 1.0 at 41 Hz and 0.7 at 238 Hz) was higher than that reported by Berndt *et al*.^22^ (0.8 at 41 Hz, and 0.3 at 100 Hz). The discrepancy might be due to differences in the experimental conditions, including preparation (*in vivo* *vs.* slice culture), temperature (normal body temperature of 37 °C *vs*. 30 °C), and/or age of animals (adult *vs*. postnatal 4–5 day). Thus a high temperature and *in vivo* mature tissue used in our experiments may result in faster and larger membrane depolarization.

An electrical recording within a 5 × 5 mm area was necessary to record these responses. Our MEMS-based mesh-ECoG has a high degree of freedom in design and is flexibly implantable over wide cortical areas, in both gyral and sulcal parts of monkey cerebrum^20^. Combination of optogenetics and the mesh-ECoG provides a potential solution to achieve high temporal resolution recording of cortically originated neural activity propagating over a wide area, which is essential to further investigate global and local neuronal interactions underlying cognitive functions.

## Materials and Methods

### Animal Preparations

This study was approved by the Niigata University Animal Experiment Committee and Niigata University Gene Modification Experiments Safety Committee, and conformed to all animal welfare laws in Japan and the NIH Guide for the Care and Use of Laboratory Animals. Nine adult Long-Evans rats (thirteen hemispheres) were used. Virus injections were conducted under sodium pentobarbiturate anesthesia (50 mg/kg body weight (b.w.), intra-peritoneal injection). In electrophysiological recording sessions the animals were anesthetized by urethane (1.0 g/kg b.w., intra-peritoneal injection) and paralyzed by *d*-tubocurarine (2.0 mg/kg b.w., intra-muscular injection) with artificial ventilation.

### AAV vector production and injection

Recombinant AAV9 vector (AAV9-hChR2-EYFP) expressing hChR2 and enhanced yellow fluorescent protein (EYFP) were produced as previously described with the exception of the viral vector concentration method^48^. The vector proviral plasmid (pAAV-CaMKIIα-hChR2(E123T/T159C)-EYFP, courtesy of Dr. K. Deisseroth) harbored hChR2-EYFP cDNA, the woodchuck hepatitis virus post-transcriptional regulatory element (WPRE) and the CaMKIIα promoter. The vector genome was packaged into the pseudotyped AAV9 capsid in AAV-293 cells (cat. no. 240073; Agilent Technologies, Santa Clara, CA). Viral vector concentration was performed by filtration (Vivaspin 20–100 K; GE healthcare, Little Chalfont, UK) and the viral titer determined by quantitative polymerase chain reaction. Four to eight weeks prior to electrophysiological recordings, rats were injected with rAAV9-hChR2-EYFP. A small craniotomy (approximately 2 mm in diameter) was made over V1 at 6 mm caudal and 4 mm lateral to bregma. In the main experiments, a total volume of 1.0 µl (2 × 10^14^ vp/mL) of viral solution was injected at an approximate depth of 1.0-mm aiming for the infragranular layers (eight hemispheres of five animals). A pipette with a Hamilton syringe was slowly lowered to depths of 1.5, 1.0, and 0.5 mm from the dural surface, and volumes of 0.4, 0.3, and 0.3 µl were injected over 8, 5, and 5 min, respectively. The pipette was left in place for an additional 5 min to allow viral diffusion. After injection, the hole above the injection site was covered with the bone fragment removed during craniotomy and fixed by dental resin, and the scalp was sutured. The sufficient injection volume and depth were determined for transgene induction into the infragraular layers by preliminary experiments. Additionally, the layer specificity was examined in two hemispheres in the main experiments using *in situ* hybridization (ISH, see *Histology*). In three additional animals, injection of viral solution was performed in the same way except that 0.2 µl of viral solution was injected at only one depth, 1.0 mm from the dural surface, for more localized transfection. Another animal was used for a control experiment in which optoelectric artifacts were recorded in the hemispheres lacking hChR2 expression.

### Electrophysiological Recordings

After 4–8 weeks of recovery, the cranial bone and dura above the injection site were widely removed to allow photostimulation and electrophysiological recordings. In the main experiments, a micro ECoG mesh (mesh-ECoG) was used for recording ECoG signals. The structure and manufacturing of the mesh-ECoG were based on those described previously^19,21,49^ with small modifications. In brief, the mesh-ECoG contained 32 electrode tips arranged in a 6 × 6 matrix with a 1 mm inter-electrode distance on a Palyrene-based thin film. The size of each electrode tip was 50 × 50 µm and the impedance was 200–300 Ω. A very thin (0.25 µm) gold layer for electrodes tips and wiring was sandwiched between two Palyrene-C layers (both 10 µm thick), and therefore the total thickness of the mesh-ECoG was approximately 20 µm, allowing high flexibility. The mesh-ECoG used in this study has multiple windows (0.7 × 0.7 mm) at inter-electrode spaces for depth electrode penetration. Photostimulation and recordings of spike activity and intracortical LFP (V1deep) were performed via an optrode, a hybrid electrode that consists of an optical fiber (Thorlabs, Inc., Newton, NJ) for photostimulation and a tungsten microelectrode (1 MΩ at 1 kHz, World Precision Instruments, Sarasota, FL) for electric recording, inserted through one of the windows (Figs 1 and 2A). The optrode was advanced vertically by a hydraulic microdrive (MO-8; Narishige, Japan). We recorded photo-evoked responses at several depths (within 0.8 to 1.5 mm from the cortical surface) along the tract, and the data recorded at the depth where the largest power at the fundamental frequency was obtained were retained for further analysis.

The mesh-ECoG was placed over the pial surface including the transgene expressing area detected using 513 nm excitation light from an LED (LED-EXTA; OptoCode, Tokyo, Japan), consequently covering almost the entire part of V1 and a part of V2. the optrode was inserted into the transgene expressing area for photostimulation and electric recording (arrows in Fig. 2A,B). The reference electrode common to both the optrode and ECoG amplifiers was inserted into the neck muscle. In the additional experiments, we employed multichannel optrodes to rule out possible effect of difference in electrodes on frequency response in the main experiment (*i.e*., tungsten microelectrode vs. mesh-ECoG) that consisted of a 16-channel silicon probe (A1x16 3mm-100; NeuroNexus, Ann Arbor, MI) and an optical fiber with a tip separated horizontally by 0.2 mm from the electrode (Supplementary Fig. S1). The sixteen recording sites were aligned along the electrode shank and separated by 100 µm.

### Photostimulation

A 473 nm blue laser (Changchun New Industries Optoelectronics, Changchun, Peoples Republic of China) was used for photostimulation. The laser power was adjusted with a neutral density filter to the minimum strength required to evoke neural responses consistently at the optrode location near to the injection site. The light power range was 0.7 to 2.2 mW at the fiber tip, comparable with previous studies (*e.g*., ≤16 mW in Ozden *et al*.^50^; 2.4 mW in Han *et al*.^38^). The laser was controlled by a dedicated stimulator (SEN-7203; Nihon-Kohden, Tokyo, Japan) for precise pulse timing. The stimulator was controlled by a custom-made program written in FreePascal/Lazarus (http://www.lazarus.freepascal.org/) using ViSaGe interface (Cambridge Research Systems, Kent, UK) running on a personal computer. The stimulation frequencies used were chosen to correspond to the theta, alpha, beta, low-gamma, high-gamma, and fast frequency bands (Supplementary Table S1). The number of pulses at each frequency within a stimulus train, and the total stimulus durations are also listed in Supplementary Table S1. The pulse width was 0.5 ms unless otherwise mentioned. Sixty stimulation trials were repeated at each stimulation site. Both eyes were covered by eye patches to prevent visual stimulation to the eyes by light migrating from the optic fiber during photostimulation. In the main experiments, 5-Hz photostimulation was performed in all the five rats and photostimulation at the higher frequencies was performed in four rats. To rule out the possible contamination by optoelectric artifacts that follow the stimulation frequency^38^, we recorded signals during photostimulation from two hemispheres of an animal lacking viral injection and from a transgene-negative area of a virus-injected hemisphere. In both control experiments, the laser power was adjusted to the maximal value used in the main experiments.

### Visual Stimulation

In some trials, we presented moving gratings as visual stimuli using ViSaGe (Cambridge Research Systems, UK) controlled by a custom-made program written in FreePascal/Lazarus. The stimulation parameters were similar to the previous report^19^ with several modification. A 15-inch LCD display (SyncMaster 541 N; Samsung, Republic of Korea) was positioned with a distance of 28.6 cm in front of the rat. The effective horizontal and vertical visual angles were 60 degree and 30 degree, respectively. The maximum brightness was 250 cd/m^2^. The eye-patch contralateral to the recording hemisphere was removed during the visual stimulation trials. The spatial and temporal frequencies of the moving gratings were 0.15 cycle/deg and 7 Hz, respectively. Stimulus duration was 800 ms and the inter-trial interval was pseudo-randomized between 1.5 and 2.0 sec.

### Data Acquisition and Analysis

Signals from the optrode and mesh-ECoG were amplified, band-pass filtered, and stored on a personal computer. In analyses of the stimulation and non-stimulation components, different bandpass filters (Butterworth, 0.7 Hz–8 kHz, 0.7 Hz–170 Hz) and sampling frequencies (20 kHz and 1 kHz) were used for unit and ECoG recordings, respectively. The intracortical LFPs (V1deep) were obtained from the optrode by digital low-pass filtering (cutoff frequency: 170 Hz, 2nd order Butterworth) and resampling at 1 kHz. In analyses of non-stimulation components and signal phase, signals from the optrode and 16 channels out of 32 ECoG channels were fed through a wide-band (0.7 Hz–8 kHz) filter and sampled at 20 kHz for fair comparison of frequency properties. To obtain spike signals, the signals from the optrode were digitally high-pass filtered (500 Hz cutoff frequency), and single unit activity was then detected using a software window discriminator with a slice level ranging 20 to 100 mV. Spikes were attributed to putative excitatory or putative inhibitory neurons based on a spike width with a criterion of 0.2 ms at half-height. Detailed descriptions were previously reported^19^.

For quantitative analyses of spectral change, we chose two ECoG recording sites (“V1surf” and “Ex-V1”) out of 32 recording sites on the mesh-ECoG for each V1deep recording site. The ECoG Near site was the most responsive site (*i.e*., the site in which the largest power at the fundamental frequency was recorded, it was also the site in which the largest absolute ERP slope was recorded) among the four sites surrounding the optrode insertion site, whereas Ex-V1 site was the most responsive site (*i.e*., the site in which the largest absolute ERP slope was recorded) site distant by more than 2.2 mm from the optrode insertion site (Fig. 1B). The stereotaxically estimated locations of Ex-V1 sites were mainly in V2 or PtP (Supplementary Fig. S2).

Frequency-domain analyses were performed by applying Fast Fourier Transformation (FFT) for every trial using a 512 ms time window sliding by 50 ms with the Hanning window function. The power spectra were averaged over trials and then divided by prestimulus power (averaged power 1.0–0.5 s prior to onset of stimulation). By this normalization, we effectively detected 238 Hz signals (both prestimulus and poststimulus signals were equally reduced by 11.6 dB using the analog filter we employed) and a slight difference between the analog low-pass filter for ECoGs and the digital low-pass filter for V1deep in the frequency properties around the cutoff frequencies was canceled out. The ECoG powers corresponding to the stimulation frequency components were calculated by extracting the FFT data within the range of the stimulation frequency or its harmonic components ± 2 Hz. To compare responses within a specific frequency band to the stimulation frequency range, we defined “Power Ratios” for V1deep, V1surf, and Ex-V1 signals as follows:

$$PowerRatio=Powe{r}_{Target}/Powe{r}_{Stim}$$

where *Power*_*Target*_ and *Power*_*Stim*_ reflect the power spectrum within the target frequency band and power spectrum within the stimulation frequency ± 2 Hz, respectively. They were averaged over trials and then normalized by prestimulus power (in dB).

For trial-by-trial time-domain comparison of low-gamma generation among V1deep, V1surf, and Ex-V1, we obtained the low-gamma band waveforms from 2048-ms data segments centered at the onset of each photostimulation period. At first, the 30–80 Hz-components were extracted from these peristimulus data using FFT, then the filtered time-domain waveforms were calculated using inverse FFT. The power spectrum within the target frequency band and power spectrum within the stimulation frequency ± 2 Hz were also filtered out. A Hanning window function was applied in FFT and filtered low-gamma waveforms were divided by the same Hanning window function to cancel out the effect of the window function. The envelopes of the filtered low-gamma waveforms were calculated using Hilbert transformation and then averaged over then whole session.

Digital filtering, window discriminating and signal averaging were performed using custom-made programs written in FreePascal/Lazarus. Digital filters were designed using the DF-design (http://momiji.i.ishikawa-nct.ac.jp/dfdesign/index_eng.shtml). FFT analyses were performed using GNU R (http://www.r-project.org/).

### Statistics

In analyses of the stimulation- and non-stimulation components, data were averaged over 16 stimulation sites (n = 1, 1, 2, 3, 1, 4, and 4 for rat#2 right, rat#3 right, rat#3 left, rat#4 right, rat#4 left, rat#5 right, and rat#5 left hemisphere, respectively). In analyses of non-stimulation components and phase shifts, data were averaged over 13 stimulation sites from four hemispheres of two rats (n = 3, 1, 5, and 4 for rat#4 right, rat #4 left, rat #5 left, and rat #5 left hemisphere, respectively), in which the same analog filter and sampling frequency were used for both icLFP and ECoG recordings for fair comparison. Results are expressed as means ± standard error of the mean (S.E.M.) unless otherwise indicated. Differences were tested using one-way ANOVA and *post-hoc* paired *t*-test with Bonferroni correction for multiple comparison or Tukey’s Honest Significant Difference with a 95% family-wise confidence level after two-way ANOVA (electrode × frequency band). For comparison of frequency-dependency between radial and tangential LFP propagation at the fundamental frequencies, we calculated attenuation of LFP during the radial and tangential transmission by subtracting V1surf from V1deep (radial) and Ex-V1 from V1surf (tangential), respectively, followed by two-way ANOVA (direction × stimulation frequency). All statistical analyses were conducted using GNU R (http://www.r-project.org/).

### Histology

After all recording sessions, the animals were perfused transcardially with 4% paraformaldehyde in 0.1 M phosphate buffer (pH 7.4) for histological analysis. To evaluate whether hChR2-YFP was selectively expressed by excitatory glutamatergic neurons, double labeling of either *GFP* and NeuN, or *vesicular glutamate transporter1* (*Vglut1*) and GFP was performed on a single section using a combination of ISH and immunohistochemistry. In brief, 20 μm thick cryosections were prepared from the hChR2-EYFP expressing rat brain samples. First, *in situ* hybridization was performed using DIG-labeled riboprobes for *GFP* or *Vglut1* as previously described^51,52^. A hChR2-EYFP expression was detected using the *GFP* probe. After obtaining dark blue-coloured ISH signals via the reaction of alkaline phosphatase and nitroblue tetrazolium (NBT)/5-Bromo-4-Chloro-3-Indolyl Phosphate (BCIP), immunohistochemical staining on the same section was performed using antibodies against NeuN (A60; Millipore, Temecula, CA; 1:200) or GFP (GF090R; Nacalai, Kyoto, Japan; 1:2,000). Immunolabeling was visualized by the reaction of peroxidase and 3′,3′-diaminobenzidine, tetrahydrochloride (DAB) as previously described^53^.

### Data availability

The datasets generated during and/or analyzed during the current study are available from the corresponding author on reasonable request.

## Electronic supplementary material

Supplementary Materials
