# Supplementary material for: Locally induced neuronal synchrony precisely propagates to specific cortical areas without rhythm distortion
Source: Sci Rep. 2018 May 16;8:7678. doi: 10.1038/s41598-018-26054-8 (PMC5956081; doi:10.1038/s41598-018-26054-8)
Supplement: Supplementary file 1 — Supplementary Materials [file 41598_2018_26054_MOESM1_ESM.pdf]

Supplementary Materials

Title
Locally induced neuronal synchrony precisely propagates to specific cortical areas without rhythm distortion

Author names
Haruo Toda, Keisuke Kawasaki, Sho Sato, Masao Horie, Kiyoshi Nakahara, Asim K. Bepari, Hirohito Sawahata, Takafumi Suzuki, Haruo Okado, Hirohide Takebayashi, Isao Hasegawa

Supplementary Figure S1

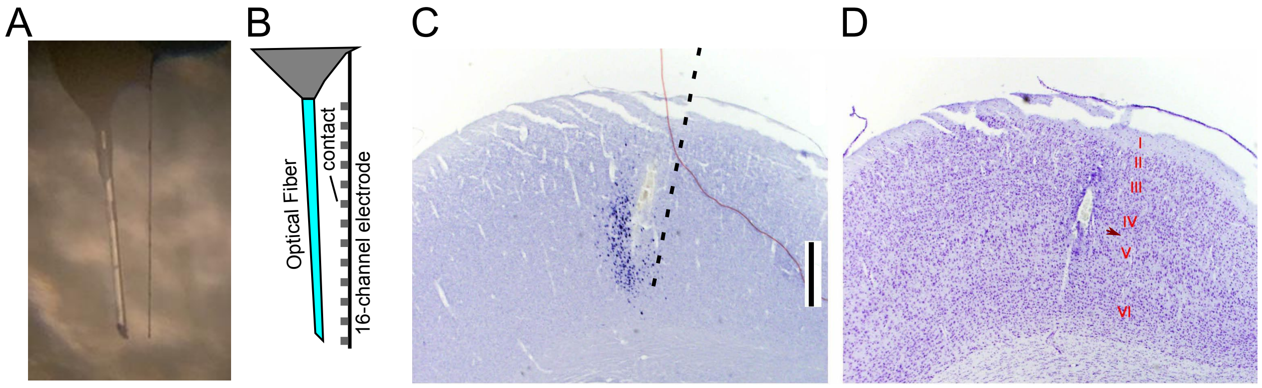

A, The multichannel optrode used in the additional experiments. *B*, A schematic illustration of the multichannel optrode. The inter-contact interval was 0.1 mm. *C*, An example of the insertion sites on a coronal section. GFP was visualized with *in situ* hybridization. Scale bar: 0.5 mm. Dotted line: approximate location of the optrode. *D*, The Nissl-stained adjacent section. Roman numbers: cortical layers. Brown arrow: the upper border of the infragranular layers.

Supplementary Figure S2

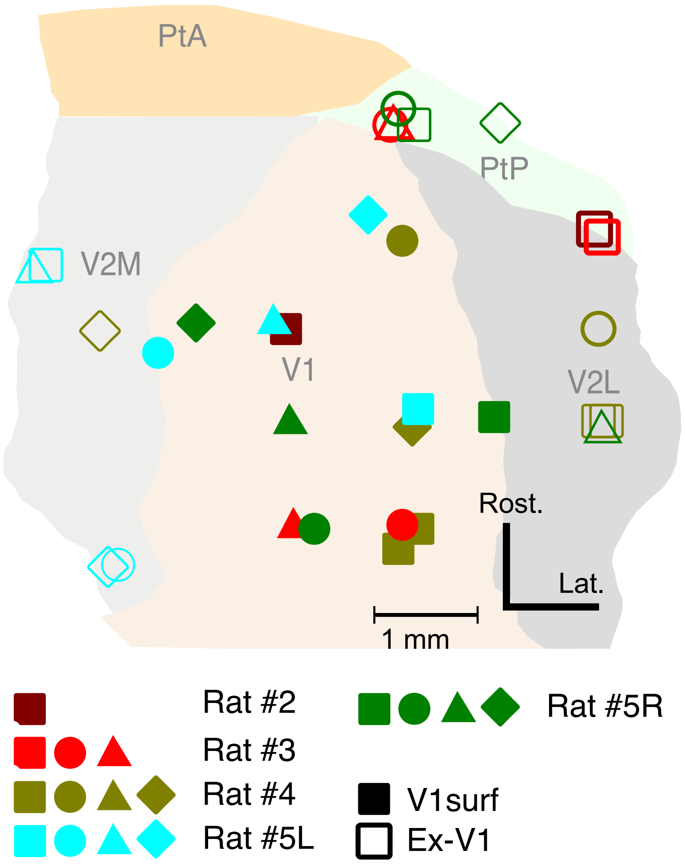

An illustration of stereotaxically estimated V1surf and corresponding Ex-V1 positions. Areal borders are adopted from Paxinos and Watson[1]. Symbol shape indicates the animal or hemisphere (in Rat #5). Filled symbols: V1surf positions. Open symbols: corresponding Ex-V1 positions. Positions on the left hemisphere are flipped horizontally and superimposed.

Supplementary Figure S3

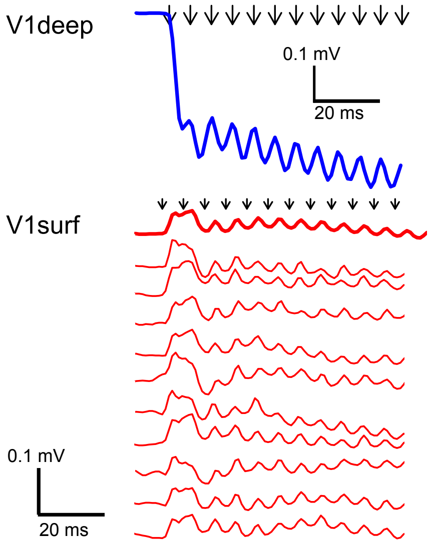

Intracortical local field potential (LFP) (V1deep) and electrocorticogram (ECoG) (V1surf) responses elicited by a 156-Hz stimulation. The top two traces are averaged V1deep (blue trace) and V1surf (thick red trace) responses simultaneously recorded from the optrode and ECoG electrode tip nearest to the optrode. Arrows indicate the timings of photo-pulses. Thin red traces represent ECoG responses in the first 10 trials from top to bottom. A “one-to-one” ECoG response to photo-pulses is evident at high-frequency stimulation. None of the traces are smoothed.

Supplementary Figure S4

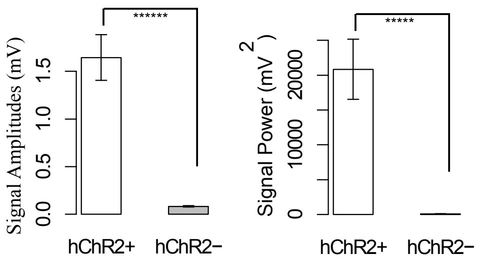

The optoelectric artifacts were significantly smaller than the photo-evoked signals. Left: The amplitudes of V1deep during 238-Hz photostimulation recorded in transgene-positive hemispheres (white) and two hemispheres of a naive animal lacking viral injection (gray). Right: Format as in the upper panel, but non-normalized powers at the fundamental frequency are shown. Data of transgene-positive hemispheres are same to **Fig.** 5*B* and **Supplementary Fig.** S6. *****: $p = 4.4 \times 10^{-5}$, ******: $p = 3.9 \times 10^{-7}$, Student's *t*-test.

Supplementary Figure S5

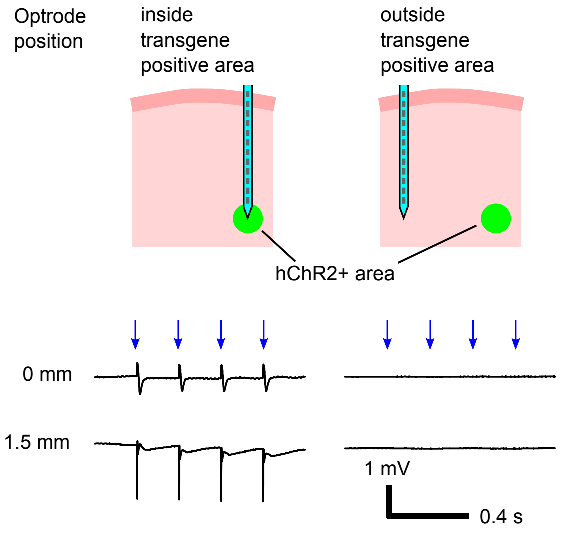

LFPs recorded via a multichannel optrode inserted at (left) the transgene-positive or (right) negative area of a vector injected rat. The data from the surface and deepest electrodes are shown. Numbers on left indicate recording depths from the cortical surface. Blue arrows indicate laser pulse timings.

Supplementary Figure S6

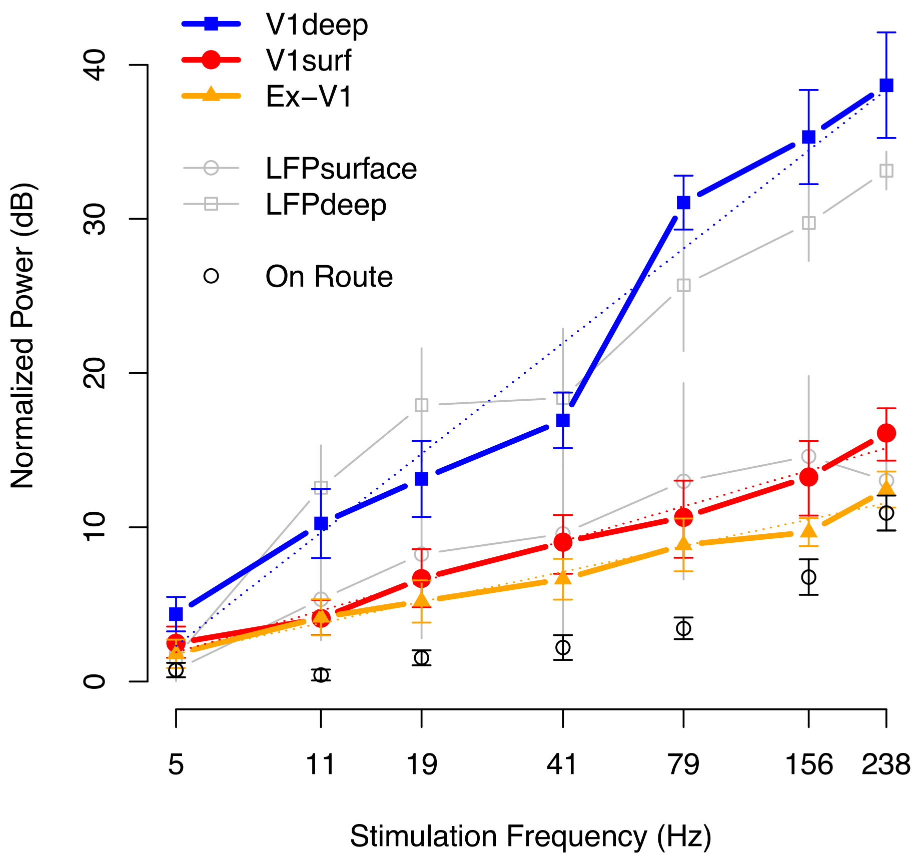

Normalized power of cortical responses at 200 ms after the onset of photostimulation as a function of frequency. The ordinate indicates V1deep (intracortical LFP at the stimulation site) or ECoG powers at the stimulation frequency divided by the pre-stimulus power. The abscissa indicates stimulation frequencies in log scale. Blue, red and orange lines represent V1deep, V1surf (ECoGs nearest to the stimulation site), and Ex-V1 (ECoGs at the outside of V1, see **Materials and Methods**), respectively. Data were averaged over 16 stimulation sites. The dotted lines colored in blue, red, and orange are regression lines for V1deep ($y = 9.34\ x - 12.7$), V1surf ($y = 3.43\ x - 3.61$), and Ex-V1 ($y = 2.46\ x - 2.08$), respectively. Slopes are in dB/oct. The thin gray lines represent the data recorded via the deep probe tip (open square) and the surface probe tip (open circle) of the multichannel optrode. n = 4. The normalized powers increased monotonically as the stimulation frequency increased, as expected from the photostimulation protocol (the energy delivered from the optical fiber was proportional to the stimulation frequency). The slope of the regression line for V1deep was significantly larger than those for V1surf and Ex-V1 (V1deep *vs*. V1surf: $p = 2.2 \times 10^{-16}$; V1deep *vs*. Ex-V1: $p = 9.0 \times 10^{-11}$; V1surf *vs*. Ex-V1: $p = 0.10$; analysis of covariance) suggesting that the ECoG responses did not increase as greatly as intracortical LFP when the stimulation frequency was high. Open circles without lines represent the data from the “on route” sites (sites between V1surf and Ex-V1).

Supplementary Figure S7
From top to bottom: bandpass-filtered low-gamma (30–80 Hz) signals at V1deep (left), V1surf (mid), and Ex-V1 (right) sites after 238-, 79-, 41-, 19-, and 11-Hz photostimulation, respectively. The red line at the top of each panel represents the envelope of bandpass-filtered signals averaged over 60 trials. The black lines below them represent example trial-by-trials bandpass-filtered signals recorded in ten successive trials. Vertical lines: onset of stimulation period. Horizontal scale bars: 0.4 s. The data were obtained in the same recording session as that in **Figure** 6*A–C*. See **Fig.** 6*C* for the data with 156-Hz stimulation.

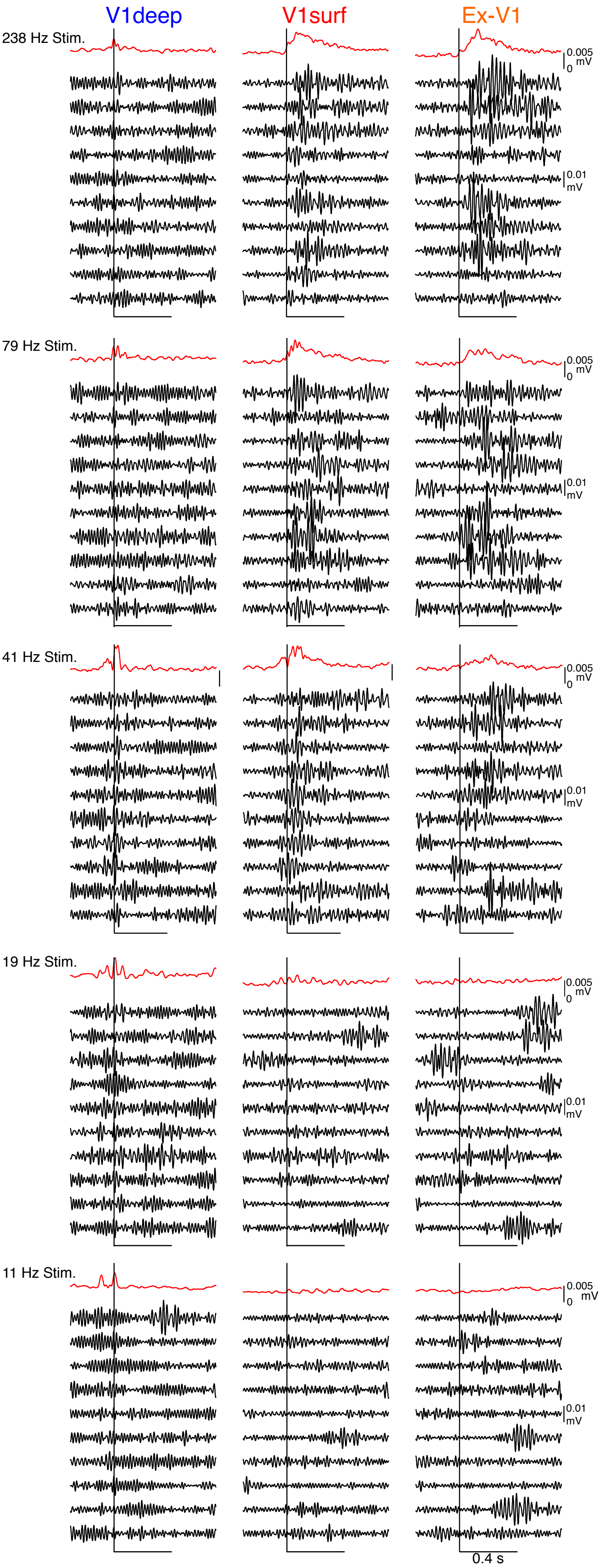

Supplementary Figure S8

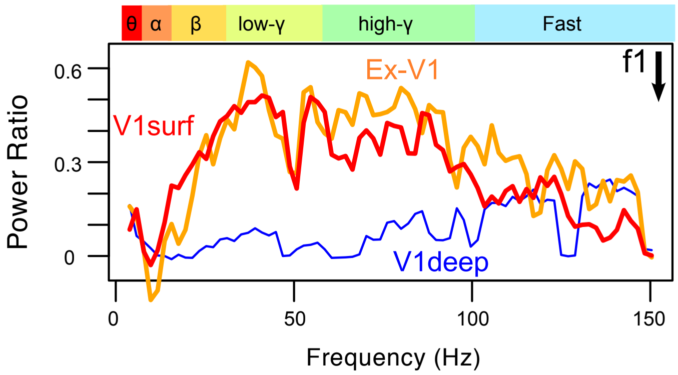

Power ratios (See **Materials and Methods**) for V1deep (blue), V1surf (red), and Ex-V1 (orange) signals at 200 ms after the stimulation onset.

Supplementary Figure S9

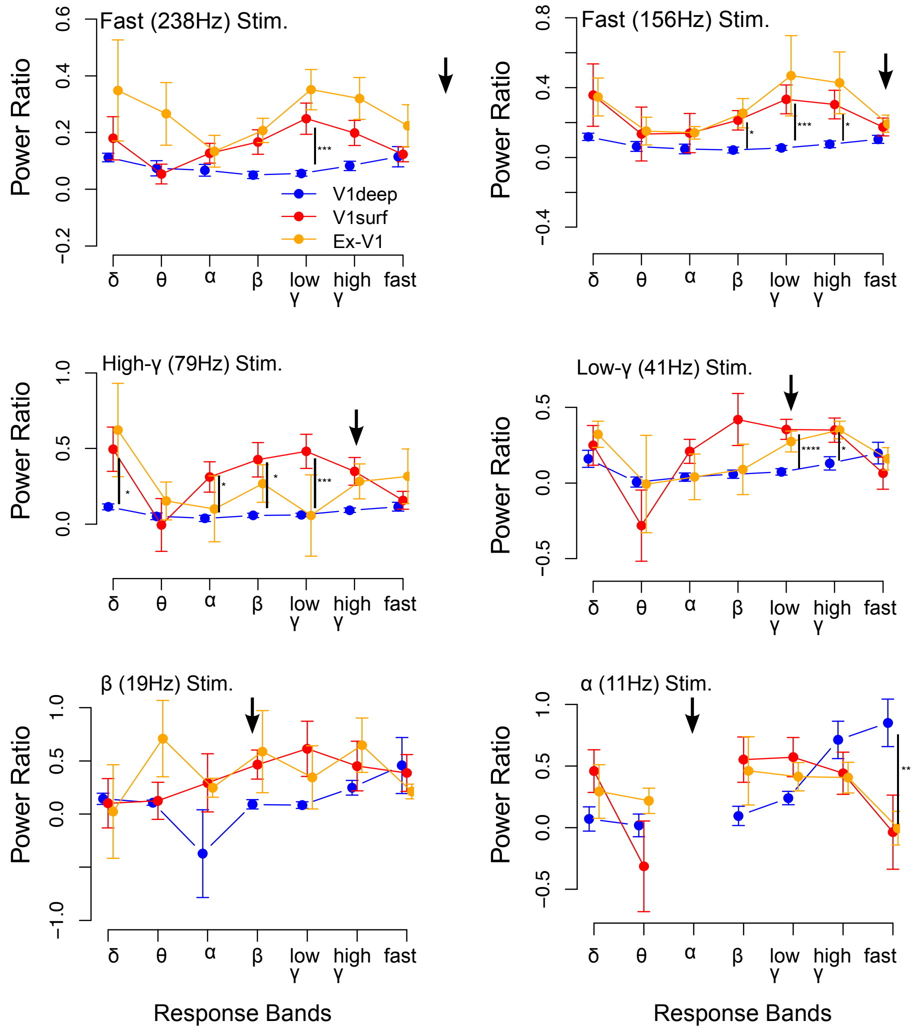

Pooled power ratios of non-stimulation components of photo-evoked cortical responses. Data were averaged over 16 stimulation sites. The stimulation frequencies were 238, 156, 79, 41, 19, and 11 Hz, respectively (indicated by downward arrows). *: $p < 0.05$, **: $p < 0.02$, ***: $p < 0.01$, ****: $p < 0.002$, *post-hoc* paired-*t* tests between V1deep and V1surf.

Supplementary Figure S10

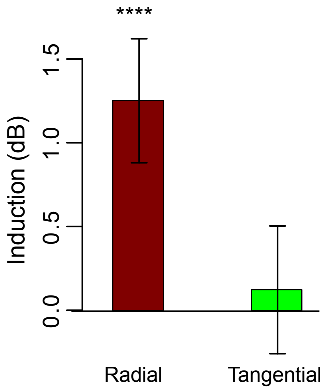

Low-gamma-band non-stimulation power induction during radial (brown) and tangential (green) propagations. Stimulation frequencies were 79, 156, and 238 Hz. ****: $p < 0.0001$, Student's *t*-test.

Supplementary Figure S11

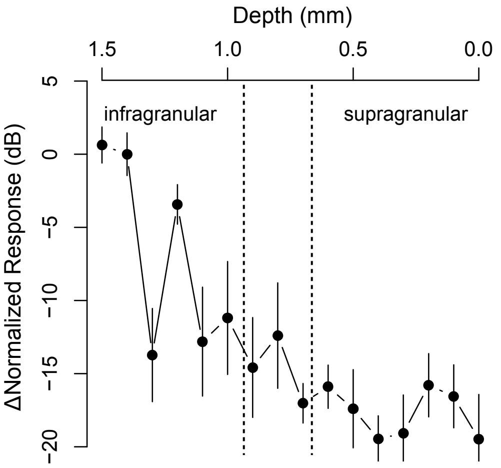

A depth profile of the power reduction at the fundamental frequency (238 Hz) components recorded by a multichannel optrode. Abscissa: depth of the probe recording site from the cortical surface. Dashed lines indicate the upper border of the infragranular layers (left) and the lower border of the supragranular layers (right).

Supplementary Table S1

The stimulation protocol for each stimulation frequency.

Band	Stimulation Frequency (Hz)	Inter-pulse-interval (ms)	Pulse Number	Duration (ms)
Theta	5.0	200	4	600
Alpha	11.0	90.9	9	727.2
Beta	19.0	52.6	15	736.4
Low-Gamma	41.0	24.4	33	780.8
High-Gamma	78.7	12.7	63	787.4
Fast	156.3	6.4	125	793.6
Fast	238.1	4.2	190	604.8

Supplementary References

1. Paxinos, G. & Watson, C. *The Rat Brain in Stereotaxic Coordinates Sixth Edition by*. **170,** (Academic Press, 2007).
